# To the Editors of the Medical and Physical Journal

**Published:** 1801-04

**Authors:** Edw. Hardman

**Affiliations:** Bolton le Moor


### *To the Editors of the Medical and Phyfical Journal.*

GENTLEMEN,

HYSTEROTOMY, or the Cefarean Section was performed upon a woman at Rochdale, about a month ago; the child was taken out alive, but is fince dead; the mother furvived till the fifth day. Three cafes of this fort have occurred in Lancafhire within little more than two years, two at Manchefter, and the one now announced at Rochdale. This is extremely fingular; for, I believe, no fuch inftance is recorded as having happened in this country for many years before. In the firft that is mentioned from Manchefter, which is the cafe of Ann Lee, the woman was in a dying ftate when fhe underwent the operation, and a putrid fœtus was extracted; but as the ftate of it could not be afcertained before birth, and as the child might have been preferved, nothing could add to the danger of the mother, the operation was unqueftionably juftifiable. In the fecond, that of Elizabeth Thompfon, the fection was made in lefs than twenty-four hours from the coming on of labour, and fhe furvived it nearly feventy-fix hours; the child lived about eighteen months. It is worthy of remark in Elizabeth Thompfon's cafe, that it appeared to the accoucheurs of the Lying-in Hofpital at Manchefter, where fhe was operated upon, that fhe did not die in confequence of the operation, but of an injury done to the uterus by the preffure of the child's head, which had taken place before the operation. It is indeed furprifing that fo little mifchief fhould be occafioned by extracting a child in this way; neverthelefs, they are gentlemen of veracity, and, in their own neighbourhood, of the firft eminence in their profeffion. I am credibly informed, that fhe was fo well the day after the operation, as to fit up in bed and fmoke a pipe of tobacco. The woman at Rochdale was feen by Mr. White, of Manchefter, foon after the operation; and at that time, as I am well informed, there was nothing in her fituation that denoted danger till the dreffings were removed from the external wound, which unfortunately appeared to be in a gangrenous ftate, and this, moft probably, was the immediate caufe of death. If women can fafely undergo this operation upon the continent of Europe fix or feven different times, of which we are well affured, why fhould practitioners be difcouraged from employing it, by its failure in the few inftances that have occurred in this country? The authority of Mr. White muft have great weight in recommending the practice, and alfo much force in doing away the the fears of thofe among us who had been deterred from practifing it by an apprehenfion of danger.

I have taken the liberty to fend you the above chiefly as an article of intelligence, which you will do me the honour of inferting in the next Number of your ufeful Journal.

I am, &c.

*Bolton le Moor, March* 14, 1801. EDW. HARDMAN.

---

### *To the Editors of the Medical and Phyfical Journal.*

GENTLEMEN,

